# Outpatient mid-urethral tissue fixation system sling for urodynamic stress urinary incontinence: 3-year surgical and quality of life results

**DOI:** 10.1007/s00192-017-3341-4

**Published:** 2017-05-12

**Authors:** Ryoko Nakamura, Masahiro Yao, Yoshiko Maeda, Akiko Fujisaki, Yuki Sekiguchi

**Affiliations:** 1 Department of Urology, Yokohama Motomachi Women’s Clinic Luna, SuzuotoBld.2F 2-96, Motomachi, Naka-ku, Yokohama, Kanagawa 231-0861 Japan; 2 Department of Urology, Yokohama City University Graduate School of Medicine, Yokohama, Japan

**Keywords:** Tissue fixation system, Stress urinary incontinence, Quality of life

## Abstract

**Introduction:**

To evaluate the clinical effectiveness and quality of life (QOL) of outpatient mid-urethral tissue fixation system sling (TFS) procedures for urodynamic stress urinary incontinence (SUI) at 3-year follow-up.

**Methods:**

We analyzed 50 mid-urethral TFS sling operations between 2007 and 2012 at Yokohama Motomachi Women’s Clinic LUNA. The primary outcome was success defined as a negative 24-h pad test, negative cough and Valsalva stress test, and no re-treatment for SUI. Secondary outcome was improvement in quality of life, which was assessed using the Incontinence Questionnaire-Short Form (ICIQ-SF) and the Incontinence Impact Questionnaire Short Form (IIQ-7). The 3-year postoperative scores were compared with baseline scores using the Wilcoxon signed rank test. A 5% two-sided significance level was used for all statistical testing.

**Results:**

All operations were carried out on an outpatient basis with no intraoperative complications. The primary cure rate result at 3-year follow-up was 90%. Median total ICIQ-SF score changed from 12 (6–20) to 0 (0–14) and median total IIQ-7 score changed from 156 (0–300) to 0 (0.00–16.7) at 3-year follow-up.

**Conclusions:**

Results show that the TFS mid-urethral sling operation is a simple, safe, effective procedure that may be done without difficulty at a freestanding clinic on an outpatient basis and favorably improves subjective QOL of the patient.

## Introduction

In Japan, we have a rapidly aging population. This brings twin problems of co-morbidities and health costs. In a 2010 editorial [1]. Macario quoted a 2005 study of 100 US hospitals in which operating room charges averaged US$62/min (range: $22 to $133/min). These costs did not include extra resources specific to the procedure. Since 2005, there have been many innovations in which surgeons seek to minimize the trauma and costs of the surgery.

One such innovation is the tissue fixation system (TFS) adjustable sling, a “micro method for cure of urinary stress incontinence,” first reported in 2005 [2]. The TFS uses two-polypropylene anchors to fix a macropore polypropylene mesh sling at the mid-urethra into the fascial structures behind the urogenital diaphragm, below the Space of Retzius. It does not require suprapubic or perineal skin perforation, and we have found it to be a safer and simpler option than existing retropubic and transobturator mid-urethral sling procedures. However, there are few reports with results using the TFS [3, 4]. In 2009, we reported 1-year results using this method [5]. The primary aim of study was to assess the effectiveness of the TFS operation at 3 years when performed under local anesthesia (LA) in an outpatient operating room setting. A secondary aim was to investigate changes in condition-specific quality of life (QOL).

## Materials and methods

### Inclusion and exclusion criteria

A total of 260 patients with urodynamically and clinically proven stress urinary incontinence (SUI) underwent TFS sling operations between 2007 and 2012 at Yokohama Motomachi Women’s Clinic LUNA. From this group, we studied 50 patients (Table 1), who met the criteria for the study. The inclusion criteria were: all patients with urodynamically proven genuine stress incontinence and a positive standardized urinary stress test, unobstructed voiding, no urodynamically demonstrated detrusor overactivity (DO), no previous urogynecological surgery, no pelvic organ prolapse, and patients who were followed for more than 3 years. The exclusion criteria were: co-morbid conditions rendering the patient unsuitable for outpatient surgery. This study was approved by the Ethics Committees of the Yokohama Motomachi Women’s Clinic LUNA in 2006 and patients provided informed consent. Patient recruitment with preoperative urodynamics and urogynecological evaluation was in accordance with International Continence Society (ICS) guidelines. Data were collected by interview and clinical examination preoperatively and 3 years postoperatively.

**Table 1:** Characteristics of 50 patients

Characteristics	Median (range) or *n* (%)
Age (years)^a^	61 (36–81)
Body mass index (kg/m^2^ ^a^	23.6 (18.2–32.9)
Parity	
0	6
1	10
2	23
3	7
4	2
5	2
Hysterectomy, *n* (%)	3 (7.40)
Urgency, *n* (%)	17 (11.1)
Intrinsic sphincteric deficiency, *n* (%)	8 (13.0)
1-h pad test (g)^a^	21.3 (0–308)
24-h pad test (g)^a^	19.0 (0–190)
Urodynamic test	
Peak urinary flow (ml/s)^a^	35.0 (14–63)
Maximum cystometric capacity (ml/s)	444 (203–692)
Post-void residual (ml/s)^a^	0 (0–73)
ALPP (cmH_2_O)^a^	85.0 (10–182)
MUCP (cmH_2_O)^a^	29.0 (1–55)

*ALPP* abdominal leak point pressure, *MUCP* maximal urethral closure pressure

^a^Median and range

### Surgery

**Fig. 1:**
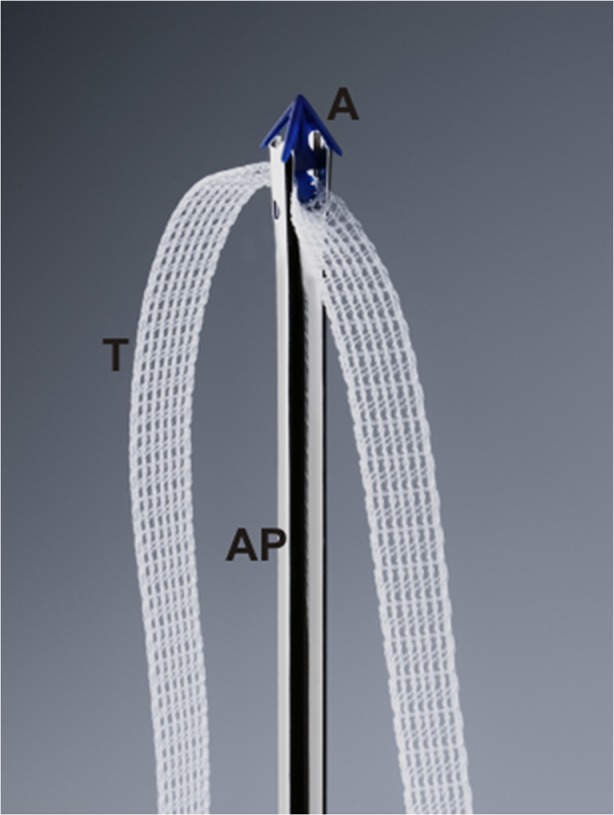
The anchor *A* sits on an applicator *AP*. A 7-mm wide macropore tape *T* passes through the base of the anchor via a one-way system that allows the tape to be tightened

**Fig. 2:**
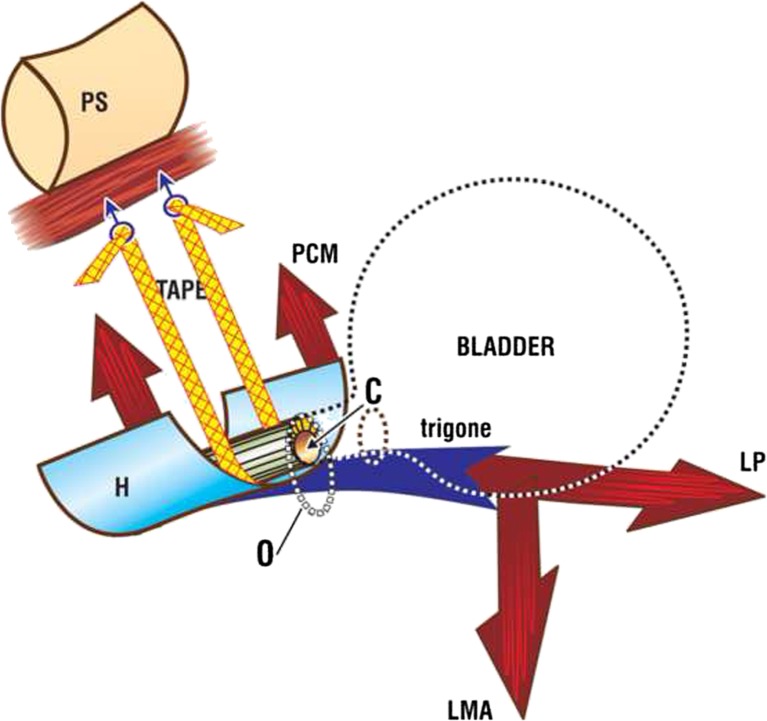
Tissue fixation system mid-urethral sling. The tape penetrates the perineal membrane to attach into the tissues immediately behind the lower end of the pubic symphysis. The tape is tightened sufficiently to prevent descent of the bladder/urethral complex on effort from the closed *C* to the open *O* position. The three-directional muscle vectors are represented by *arrows*. *PCM* coccygeus muscle, *LP* levator plate, *LMA* conjoint longitudinal muscle of the anus *H* suburethral vagina (hammock), *PS* pubic symphsis. [6]. Published by permission of the author

**Fig. 3:**
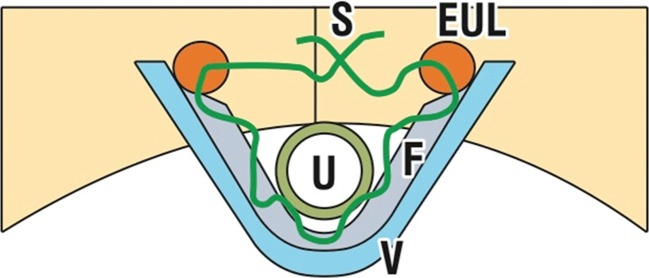
Tightening of the distal supports of the urethra with a 20 vicryl suture *S*, which passes through the musculoelastic layer of the vagina to tighten it toward the external urethral ligament (*EUL*). The EUL attaches the external meatus of the urethra *U* to the anterior surface of the pubic bone *V* vagina [6]. Published by permission of the author

The TFS consists of an 11x4-mm, four-pronged anchor with a one-way tensioning system at its base through which passes a 7-mm lightweight macropore polypropylene tape (Fig. 1) [6]. The technique is similar to the first part of the TVT procedure. The aim is to create a channel into the tissues in the region of the origin of the pubo-urethral ligament immediately behind the inferior surface of the pubic bone, to insert anchors into the channels made, and to tighten the tape sufficiently to restore the closure mechanism as indicated in Fig. 2. A no. 18 Foley catheter is inserted into the bladder. The vagina and deeper tissues are infiltrated with saline containing 1% xylocaine. Using Allis forceps, the vagina is grasped 0.5 cm below the meatus and immediately behind the mid-urethra. Under tension, a full-thickness incision is made in the vagina between the two forceps. Metzenbaum scissors are used to create a tunnel. When perineal membrane resistance is felt, the tip of the scissors is guarded and the perineal membrane and the tissues behind it are penetrated to a depth of 1.5–2 cm. The scissors are now removed and an applicator with an anchor on its tip is inserted into the tissues. Again, resistance is felt. The applicator is guarded and penetrates the tissues behind the pubic bone for 1.5–2 cm, at the origins of the pubo-urethral ligament. The anchor is released by pressing a button on the handle and the applicator is now removed. The insertion is repeated on the contralateral side. To avoid constriction by the tape, a no. 8 Hegar dilator is inserted into the urethra. The applicator is now held firmly with one hand while the other grasps the free end of the tape. The free end is pulled until resistance is felt. At this point, the urethra is inspected to ensure that the tape touches but does not indent the urethra [6]. A cystoscopy is performed. The patient is asked to cough. If there is a leakage, the Hegar is re-inserted and the tape is tightened again. If there is no leakage, the applicator is removed and the free end of the tape is cut. The sub-urethral vagina was then sutured to restore the anatomy of the distal (“hammock”) closure mechanism as in Fig. 3, with a “tobacco pouch” suture using 00 vicryl: first, the external ligament lateral to the external meatus is penetrated, the musculofascial layer of the vagina on one side, then the other side, and finally, the contralateral external ligament is sutured; the suture is gently tied, but not tightly [6].

### Outcome measures

We used two condition-specific measures of QOL to assess the change in QOL after the operation: the Incontinence Impact Questionnaire Short Form (IIQ-7) and the Incontinence Questionnaire-Short Form (ICIQ-SF). Overall treatment success was defined as no self-reported SUI symptoms, negative 24-h pad test (defined as less than 3 g), negative cough and Valsalva stress test, and no re-treatment for SUI including behavioral, pharmacological or surgical therapies. Treatment failure was considered to be failure in any one of the outcome criteria listed previously. Continuous variables are reported with average and range, whereas categorical variables are presented with frequency and percentage.

### Analysis of data

The computer program SPSS for Windows version was used for statistical analysis. Values are presented as median (range). The 3-year postoperative scores were compared with baseline scores using the Wilcoxon signed rank test. A 5% two-sided significance level was used for all statistical testing.

## Results

A total of 50 patients with SUI completed a minimum of a 3-year follow-up. Demographic and baseline characteristics are shown in Table 1. Median age was 61 (36–80) years and median body mass index was 23.6 (18.2–32.9) kg/m^2^. Median 1-h pad test was 17.5 (0–290) g and median 24-h pad test was 20.0 (0–705) g.

### Surgical results

The operations were performed by two surgeons using local anesthesia and patients also received 2.5 mg midazolam. All operations were done on an outpatient basis. Median operative time was 20 (14–55) min, median blood loss was 5.0 (0–100) ml and median clinic stay time was 4.70 (2.25–6.29) h. There were no intraoperative complications and only minimal postoperative pain, which was treated with paracetamol. There was no case of damage to the bladder or to nerves or vessels during the operation. No transfusion was required. Patients were discharged home after the first urination or 8 h postoperatively. Five patients needed indwelling catheter, and all 5 patients voided without difficulty within 2 days. Of 16 patients taking anticholinergic drugs for their symptoms of urge urinary incontinence preoperatively, 8 were cured of their urge, whereas the other 8 patients continued their treatment. One patient developed de novo urge incontinence postoperatively and required an anticholinergic drug.

### Three-year postoperative results

Three-year postoperative success was noted in 45 patients (90%). There were 5 patients in whom treatment was thought to have failed according to our criteria. These 5 patients requested re-operation, also with TFS, and all of them were continent after the reoperation. There were no tape infections or erosions and no tape/anchor extrusions for 3 years after the operation. We diagnosed intrinsic sphincteric deficiency (ISD) if an abdominal leak point pressure (ALPP) was less than or equal to 60 cmH_2_O. We had 6 patients with ISD. The median ALPP of these ISD patients was 42 (28–58) cmH_2_O. Five of these patients were continent after the TFS operation and the other requested reoperation for recurrence.

### Impact on QOL

Considering QOL (Tables 2 and 3), median total ICIQ-SF score changed from 12 (6–20) to 0 (0–14) and median total IIQ-7 score changed from 159 (0.00–300) to 0.00 (0.00–167) on assessment 3 years after surgery. All domains of the ICIQ-SF and IIQ-7 significantly improved at 3-year follow-up.

**Table 2:** Comparison of mean Incontinence Questionnaire-Short Form (ICIQ-SF) scale score pre- and 3 years postoperatively

	Preoperatively	Three years postoperatively	*p* value
Total	12 (6–20)	0 (0–14)	< 0.00001
Frequency	4 (1–5)	0 (0–4)	< 0.0001
Quantity	2 (2–6)	0 (0–4)	< 0.00001
Quality of life	5 (1–9)	0 (0–4)	< 0.0001

**Table 3:** Comparison of Incontinence Impact Questionnaire-Short Form (IIQ-7) scores between pre- and 3 years postoperatively

	Preoperatively	Three years postoperatively	*p* value
Total	156 (0.00–300)	0.00 (0.00–16.7)	< 0.0001
Physical activity	50.0 (0.00–100)	0.00 (0.00–16.7)	< 0.0001
Travel	33.3 (0.00–100)	0.00 (0.00–16.7)	< 0.0001
Social	33.3 (0.00–100)	0.00 (0.00–50.0)	< 0.0001
Emotional	33.3 (0.00–100)	0.00 (0.00–50.0)	< 0.0001

## Discussion

This is our second report of successful mid-urethral sling surgery for SUI safely performed under LA using the TFS minisling operation in a freestanding clinic. Our clinic is a conventional freestanding clinic with a small (but adequate) operating room and no overnight facilities. The capital and running costs are clearly miniscule compared with such operations performed in a major hospital facility. Against a background of rising costs and an aging population, the high 3-year success rate (90%), minimal postoperative pain, and same-day discharge are powerful arguments for these operations to be performed in a freestanding clinic using our methodology. A further benefit was how well received this method was with the patients in that they did not need to go into hospital.

Sivaslioglu et al. reported a significantly higher subjective cure for TFS, a retropubic tensioned minisling, 89% compared with 78% for transobturator tape (TOT) at 5 years [3]. In the first report of a minisling in the literature (*n* = 36), Petros et al. reported 83% cure of SUI on an intention-to-treat basis. They reported cure of 9 patients with ISD and 80% improvement in a 10th patient [2]. Kocjancic et al. [7] reported on 113 subjects who were implanted with the Altis (tensioned) sling. One hundred and one had efficacy data at baseline and at 12 months. 90.1% at 12 months achieved a 50% or greater reduction in pad weight. In an RCT, Lee et al. reported equivalent results for the mini-Arc, an untensioned minisling, compared with a TOT sling, >90% at 6 months [8]. However, these results are not typical for untensioned minislings. In a 2-year RCT of the untensioned TVT Secur minisling (*n* = 56) vs TOT, (*n* = 66), Bianchi-Ferraro et al. reported subjective cure rates of 75.7% and 80.3% respectively [9].

Basu and Duckett [10] reported poor results for a single incision minisling at 3 years compared with the TVT. In the single-incision sling group, the failure rate increased from 40.5% at 6 months to 52.6% at 3 years with corresponding figures of 3 to 9% in the retropubic mid-urethral sling group. Our 3-year postoperative data were equivalent to that of the Basu’s TVT arm, and other TVT results [11]. We explain this discrepancy as follows: the TFS is identical to the TVT in principle and similar in methodology, albeit simpler and safer. The TFS is retropubic and mid-urethral. It uses the same artificial neoligament principle to repair damaged pubo-urethral ligaments (PULs). It is first inserted below the middle part of the urethra and tensioned over a no. 8 Hegar dilator as a separate movement to a precision of 1 mm. In contrast to the TVT, we used a shorter sling; the tape anchors the base of the urethro-pelvic ligaments to the origin of the PULs and neighboring musculofascial structures [2]. Because the TFS is inserted below the retropubic space, it is virtually impossible to injure the bladder, intestine or major blood vessels, which are major potential problems with the TVT. Avoiding skin perforation and minimizing tissue trauma also minimize postoperative pain. All this allowed the TFS mid-urethral sling operations to be performed using LA on an outpatient basis. In contrast to the TFS and TVT, the Miniarc sling has to perform insertion and tightening simultaneously. The tapes compress the elastic tissues of the urethra and paraurethral tissues. Allowance has to somehow be made for elastic restoration of the tissues. This can only be estimated. We believe that tightening over a no. 8 Hegar dilator minimizes postoperative urinary retention. We attribute cure of ISD in this and in our 2009 study to the precision of the tightening system at the base of the anchor.

De novo urge symptoms have been a problem with both the traditional and sling incontinence operation. Segal et al. reported a de novo urgency incidence of 4.3% and de novo urge incontinence of 9.1% after operation in pure SUI patients [12]. In our study, only one patient (2.0%) developed de novo urgency. We attribute this low rate to the accurate tightening system that prevents the over-elevation that we consider a major cause of de novo urgency.

**Fig. 4:**
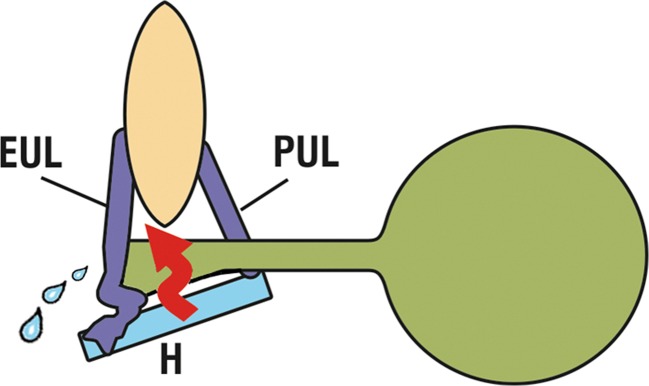
A loose EUL vagina (*H*) may lengthen and weaken the pubococcygeus muscle closure forces (*arrow*) [6]. Published by permission of the author

We believe that the high cure rate achieved for SUI may be due not only to the precise tensioning mechanism, but also to the great care that we took to restore the anatomy of the distal hammock closure mechanism. Figure 4 schematically shows how a loose external ligament/vagina may invalidate distal urethral closure by effectively lengthening the pubococcygeus muscle (arrow), thus weakening its closure force [13].

The importance of the distal urethral closure mechanism (Fig. 4) was recognized in the original mid-urethral sling methodology, which recommended suburethral vaginal tightening in cases where it was loose [14].

Although not life-threatening, SUI has a significant impact on a patient’s QOL. The International Continence Society has recommended the inclusion of health-related QOL as an outcome in clinical studies of urinary incontinence to complement clinical measures [15]. We used the ICIQ-SF and IIQ-7 to evaluate QOL. The ICIQ-SF is a brief three-scored and one-unscored self-diagnostic item that assesses the prevalence, frequency, and volume of leakage, in addition to the impact on QOL [16, 17]. In this study, the total ICIQ-SF score improved from 12 to 0 at 3 years after the operation. Meschia et al. used the ICIQ to assess improvements in QOL, showing 80–90% improvements in overall scores [18]. IIQ-7 is a short form of IIQ. Seven questions measure the effect of UI on daily activities within four subscales of physical activity, travel, social/relationships, and emotional health. Each of the subscales ranges from 0 to 100. The subscale scores are summed for a total score from zero to 400, with higher scores representing a more negative impact on QOL, unlike the ICIQ, which includes frequency and severity of SUI of nearly half of the total score. In this study, total IIQ-7 score decreased from 157 to zero. This improvement is consistent with other reports [18–20].

Six patients had ISD and 5 of them were cured. In a previous publication [5], 15 out of 44 patients (34.4%) had ISD with the same cure rate as non-ISD patients (90.9%). Although the numbers in either publication are too low for any valid conclusion, it is our belief that precise adjustability of the sling and attention to restoration of the distal closure mechanism (Fig. 4) are important surgical principles to be observed for treatment of this difficult condition.

The strength of this study was the demonstration that using the retropubic TFS adjustable sling, high cure rates can be achieved over 3 years in an outpatient facility. Potentially, large savings with no compromise to safety are possible by uptake of this method for treating SUI.

One limitation was that we did not quantify costs. Another was the retrospective nature of the study.

## Conclusions

The TFS procedure can be considered a simple, safe and cost-effective procedure for the treatment of SUI that may be performed without difficulty on an outpatient basis with major potential cost savings for the community.

## Abbreviations

ALPP: Abdominal leak point pressure
DO: Detrusor overactivity
EUL: External urethral ligament
ICIQ-SF: Incontinence Questionnaire-Short Form
ICS: Incontinence Society
IIQ-7: Incontinence Impact Questionnaire Short Form
ISD: Intrinsic sphincter deficiency
LA: Local anesthesia
MUCP: Maximum urethral closure pressure
OAB: Overactive bladder
PUL: Pubo-urethral ligament
TFS: Tissue fixation system
TOT: Trans-obturator tape
TVT: Tension-free vaginal tape
